# Treating neuromuscular diseases: unveiling gene therapy breakthroughs and pioneering future applications

**DOI:** 10.1186/s12929-025-01123-z

**Published:** 2025-02-21

**Authors:** Yu-Fu Wu, Jun-An Chen, Yuh-Jyh Jong

**Affiliations:** 1 Institute of Molecular Biology, Academia Sinica, Taipei, Taiwan; 2 Neuroscience Program of Academia Sinica, Academia Sinica, Taipei, Taiwan; 3 Graduate Institute of Clinical Medicine, College of Medicine, Kaohsiung Medical University, Kaohsiung, Taiwan; 4 Department of Pediatrics, Division of Pediatric Neurology, and Translational Research Center of Neuromuscular Diseases, Kaohsiung Medical University Hospital, Kaohsiung Medical University, Kaohsiung, Taiwan; 5 Department of Biological Science and Technology, National Yang Ming Chiao Tung University, Hsinchu, Taiwan

**Keywords:** NMD, Motor neuron, Gene therapy, ASO, SMA, DMD, AAV

## Abstract

In this review, we highlight recent advancements in adeno-associated virus (AAV)-based gene therapy for genetic neuromuscular diseases (NMDs), focusing on spinal muscular atrophy (SMA) and Duchenne muscular dystrophy (DMD). We discuss the current FDA-approved gene therapies for NMDs and provide updates on preclinical studies that demonstrate the potential of various AAV-based gene therapies to reduce SMA severity and serve as effective treatments for DMD. Additionally, we explore the transformative impact of CRISPR/Cas9 technology on the future of gene therapy for NMDs. Despite these encouraging developments, further research is required to identify robust biomarkers that can guide treatment decisions and predict outcomes. Overall, these pioneering advancements in AAV-based gene therapy lay the groundwork for future efforts aimed at curing genetic NMDs and offer a roadmap for developing gene therapies for other neurodegenerative diseases.

## Background

Neuromuscular diseases (NMDs) encompass all disorders caused by abnormalities in motor units, affecting the motor neurons of the spinal cord, neuromuscular junctions, or muscle tissues. In addition to NMDs caused by infection, autoimmunity, drugs or environmental chemicals, most other NMDs are hereditary degenerative diseases. Currently, more than 1216 genetic NMDs, involving at least 686 pathogenic genes and proteins (including 78 mitochondrial genes), have been identified. These conditions are often classified as rare diseases, affecting approximately 1 in 2000 people worldwide [1, 2]. Over the past decade, rapid developments and groundbreaking advancements in gene sequencing technologies have made molecular diagnoses of genetic NMDs more accessible, allowing for precise disease-modifying therapies (DMTs) that target the root causes of these conditions, apart from addressing their symptoms [3, 4].

Since 2016, the Federal Drug Administration (FDA) of the United States of America has approved various gene therapies as DMTs to treat genetic NMDs, providing the opportunity to change the fate of individuals severely affected by these conditions. Gene therapy is a cutting-edge medical technique aimed at treating or preventing diseases by directly or indirectly modifying the genetic material within a patient's cells. This approach holds the potential to address a wide range of genetic disorders, cancers, infectious diseases, and other pathologies that currently have limited treatment options [5]. A broad sense of gene therapies for specific NMDs can either be delivered via viral vectors or directly administered in the form of antisense oligonucleotides (ASO) or small molecule drugs. Nowadays, gene therapy is usually referred as viral vector-mediated gene/small RNA delivery as a means to treat NMDs. Based on the mechanism of action, a wide range of gene therapies can be categorized into the following four main strategies (see also the summary in Table 1):

1. Gene replacement therapy: This approach aims to correct monogenic diseases by providing a functional copy of the defective gene, thereby enabling normal protein production. For example, Hemophilia B is a blood clotting disorder caused by mutations in the F9 gene, which encodes the clotting factor IX. Gene therapy for Hemophilia B uses an AAV5 vector to deliver a functional copy of the *F9* gene into liver cells, allowing patients to achieve sustained production of functional factor IX and thereby reducing the frequency of bleeding episodes and the need for regular factor IX infusions [6]. Other diseases being tackled by this approach are spinal muscular atrophy (SMA) (onasemnogene abeparvovec) [7], Duchenne muscular dystrophy (DMD) (delandistrogene moxeparvovec) [8], and sickle cell disease (lovotibeglogeno cutotemce) [9].
2. Gene addition: This approach, typically employed for cancer, infectious diseases, and other complex disorders, involves supplementing the patient with therapeutic genes that target specific aspects of the disease mechanism. In Bacillus Calmette-Guérin (BCG)-unresponsive nonmuscle-invasive bladder cancer, the first intravesical gene therapy uses a non-replicating recombinant adenovirus vector to deliver the human *Interferon alpha-2b* gene to urothelial cells. This therapy elicits direct impacts such as cell death and mediation of an antiangiogenic effect. Indirectly, it initiates immunomodulation of the innate and adaptive immune responses [10].
3. Gene knockdown by RNA interference (RNAi) or regulating messenger RNA (mRNA) splicing by ASO or small molecules: This method uses small interfering RNA (siRNA) molecules to degrade specific mRNAs, thereby reducing the production of disease-causing proteins. For instance, familial amyloid polyneuropathy (FAP) is a hereditary disorder caused by mutations in the *transthyretin* (*TTR*) gene, leading to accumulations of misfolded TTR proteins and amyloid deposits in the peripheral nerves and other tissues. Gene knockdown therapy for FAP involves using siRNA molecules encapsulated in lipid nanoparticles to target and degrade *TTR* mRNA in the liver, reducing the production of both mutant and wild-type TTR proteins. Consequently, serum levels of TTR are significantly reduced and neurological function and patient quality of life are improved [11]. Other diseases being targeted by this approach are SMA (Spinraza, ASO [12]; Evrysdi, small molecule [13]), amyotrophic lateral sclerosis (ALS) (Tofersen, ASO) [14], and DMD (Etepliersen, exon 51 skipping, ASO [15]; Golodirsen, exon 53 skipping, ASO [16]; Viltolarsen, exon 53 skipping, ASO [17]; and Casimersen, exon 45 skipping, ASO [18]).
4. Gene correction or editing: This strategy involves introducing targeted changes in the host genome to correct genetic mutations or modify gene expression. For example, sickle cell disease (SCD) and transfusion-dependent β-thalassemia (TDT) are severe monogenic diseases. BCL11A is a transcription factor that represses γ-globin and fetal hemoglobin (HbF) in erythroid cells. Gene editing approaches for both diseases aim to induce HbF expression to alleviate symptoms. By using CRISPR-Cas9 to target the BCL11A erythroid-specific enhancer in CD34^+^ hematopoietic stem and progenitor cells from healthy donors, ~ 80% allele modification without off-target effects has been achieved. In a clinical report, two patients, one suffering TDT and the other SCD, received autologous CD34^+^ cells edited in the same way after myeloablation. More than a year later, both patients showed high levels of allelic editing, increased pancellular fetal hemoglobin, transfusion independence and, for the SCD patient, elimination of vaso-occlusive episodes [19].

**Table 1:** The four main strategies of gene therapy illustrated by FDA-approved drugs

Mechanism	Disease	Target gene	Route	Drug [brand name, year of FDA approval]	Refs.
Gene replacement therapy	Hemophilia B	*F9E*	i.v.	Etranacogene dezaparvovec [Hemgenix, 2022]	[6]
	Spinal muscular atrophy	*SMN1*	i.v.	Onasemnogene abeparvovec [Zolgensma, 2019]	[7]
	Duchenne muscular dystrophy	*DMD*	i.v.	Delandistrogene moxeparvovec [Elevidys, 2023, 2024]	[8]
	Sickle cell disease	*HBB*	i.v.	Lovotibeglogeno cutotemce [Lyfgenia, 2023]	[9]
Gene addition	BCG-unresponsive nonmuscle-invasive bladder cancer	*IFNα2b*	i.ves.	Nadofaragene firadenovec [Adstiladrin, 2022]	[10]
Gene knockdown (RNAi, ASO, small molecule)	Familial amyloid polyneuropathy	*TTR*	i.v.	Patisiran [Onpattro, RNAi, 2018]	[11]
	Spinal muscular atrophy	*SMN1*	i.t.	Nusinersen [Spinraza, ASO, 2016]	[12]
		*SMN1*	Oral	Risdiplam [Evrysdi, oral small molecule, 2020]	[13]
	Amyotrophic lateral sclerosis	*SOD1*	i.t.	Tofersen [Qalsody, ASO, 2023]	[14]
	Duchenne muscular dystrophy	*DMD*	i.v.	[Etepliersen, ASO, 2016]	[15]
		*DMD*	i.v.	[Golodirsen, ASO, 2019]	[16]
		*DMD*	i.v.	[Viltolarsen, ASO, 2020]	[17]
		*DMD*	i.v.	[Casimersen, ASO, 2021]	[18]
Gene editing#	Sickle cell disease	*HBB*	i.v.	Exagamglogene autotemcel# [Casgevy, 2023]	[19]
	Transfusion-dependent beta thalassemia	*HBB*	i.v.	Exagamglogene autotemcel# [Casgevy, 2024]	[19]

RNAi: RNA interference; ASO: antisense oligonucleotide; #: cell-based gene therapy treatment utilizing CRISPR/Cas9 gene editing technology; i.v.: intravenous; i.t.: intrathecal; i.ves.: intravesical, Ref.: reference

To administer gene therapies via viral vectors, AAV is commonly used to deliver the desired gene to a particular target [20]. AAV delivers genes by entering cells via endosomes and yet, importantly, the delivered genes are not integrated into the genome. Upon endosomal rupture, the therapeutic DNA enters the cell nucleus as a double-stranded molecule and it is rendered ready for transcription by forming a circular episome [21]. Several characteristics of AAVs make them amenable as a platform for the production of recombinant vectors used in gene therapy, such as their lack of pathogenicity, defective replication, non-genome-integrating behavior, ability to establish long-term transgenic expression, and multiple serotypes permitting liver targeting [20, 22–24].

The current success of AAV-based gene therapies for genetic NMDs is aptly exemplified by two well-characterized diseases, i.e., SMA and DMD. Therefore, in the following sections of this mini-review, we update on current progress of clinical trials for SMA and DMD gene therapies, as well as several preclinical studies on AAV-mediated treatments. Then, we discuss current progress and future potential applications of pioneering in vivo base-editing approaches. Finally, we briefly summarize the potential applications of NMD biomarkers as an accessory approach to advance novel gene therapies.

## Successful gene therapy of SMA, complemented by ASO-based and small molecule therapies for patients of all ages

SMA is an autosomal recessive neurodegenerative disorder manifesting as degeneration of spinal motor neurons, with concomitant muscle atrophy and weakness. This disorder arises from an autosomal recessive mutation in or deletion of the *Survival Motor Neuron 1* (*SMN1*) gene [25]. SMA patients possessing bi-allelic mutations in the *SMN1* gene exhibit symptoms such as weakness in the muscles that control movement, breathing, and swallowing. SMA is one of the most prevalent genetic disorders affecting young children, and it is a major cause of death in infancy. Evolutionary conservation of the *SMN1* gene across metazoans highlights its essential role, with *SMN1* loss-of-function typically resulting in embryonic lethality. Notably, the human genome harbors a unique hypomorphic paralog, *SMN2*, that shares 99% sequence identity with *SMN1* but that is characterized by a C-to-T nucleotide variant in exon 7 that often results in exon exclusion. Consequently, only ~ 10% of *SMN2* transcripts are complete and translated into functional SMN protein. This restricted production of SMN protein from the *SMN2* transcript is pivotal for the survival of SMA patients, with *SMN2* gene copy number being a key genetic determinant of SMA severity [26, 27]. However, recent research has shown that SMA can also affect various other tissues and organ systems, which is why it is increasingly being referred to as a systemic disease [28–30].

Restoring SMN protein levels in spinal motor neurons has been regarded as the most straightforward approach to curing SMA and the one with the greatest potential, but an effective treatment was not available until a few years back. As levels of complete *SMN2* transcripts are generally inversely correlated with SMA severity, the development of SMA therapies began by screening for modifiers of *SMN2* mRNA splicing to facilitate inclusion of exon 7, thereby increasing the production of full-length *SMN2* transcripts. After years of basic research followed by clinical trials, two splicing modifiers have been approved by both the U.S. FDA and European Medicines Agency (EMA) to treat SMA: nusinersen, an antisense oligonucleotide (ASO) drug (approved in 2016 and 2017 by the FDA and EMA, respectively) [12]; and risdiplam, a small molecule oral drug (approved in 2020 and 2021, respectively) [13].

Nusinersen (or ASO-10–27) is an ASO that binds to the intron downstream of exon 7 in *SMN2* pre-mRNA at an intronic splicing silencer, thus promoting exon 7 inclusion and ultimately enabling production of more functional SMN protein. A preclinical proof-of-principal study using SMNΔ7 (Smn^−/−^, SMN2^+/+^, SMNΔ7^+/+^) mice, a mouse model of severe SMA, achieved increased SMN protein levels in spinal motor neurons via single intracerebroventricular (i.c.v.) administration of nusinersen. This treatment directed at the central nervous system (CNS) was sufficient to ameliorate motor neuron loss in the spinal cord of the mice, improve myofiber size and NMJ morphology, enhance motor function, and promoted survival [31]. In addition, peripheral administration of nusinersen via subcutaneous injection provided additional benefits to the SMNΔ7 mice apart from neuromuscular rescue, including improved heart histology. Since nusinersen cannot penetrate the blood brain barrier, these outcomes highlight the potential systemic significance of restoring SMN protein levels beyond the CNS [32]. Clinically, both infants and children receiving intrathecal injections of nusinersen display significant and clinically meaningful improvements in motor function, with infants being more likely to survive compared to control groups (ENDEAR, NCT02193074 [12]; CHERISH, NCT02292537 [33]).

In contrast, risdiplam is a small molecule drug that also promotes inclusion of exon 7 in *SMN2* transcripts. It was discovered through a serial process of chemical screening and optimization. Importantly, risdiplam and its related compounds are delivered orally and can penetrate into various tissues, including the brain, spinal cord, and muscle, leading to increased SMN levels in the CNS and muscle of mouse models of both mild (C/C-allele) and severe forms (SMNΔ7) of SMA. Administration of risdiplam to SMNΔ7 mice has been found to improve their motor function, preserve neuromuscular connectivity, and extend survival [34–37]. Clinically, meaningful and significant improvements in event-free survival, attainment of motor milestones, and enhanced motor functions have been reported from clinical trials on type 1 SMA (FIREFISH, NCT02913482) and type 2 and 3 SMA (SUNFISH, NCT02908685) patients after certain issues regarding safety and target-specificity were alleviated [35–38].

While both ASO and small molecule seem to be effective, and this once-incurable disease can now benefit from a choice of different life-saving therapies, there are still some limitations in real clinical settings. For instance, exorbitant costs often hinder the feasibility of currently available therapeutics. In the U.S., nusinersen treatment costs US$750,000 in the first year, including the loading doses, and then US$375,000 for every subsequent year. Risdiplam costs US$100,000–340,000 per year [39]. In addition, as lifelong repeat dosing is necessary for the aforementioned SMA treatments, which is stressful for both clinicians and patients, a one-time treatment that permanently cures the disease is clearly more desirable. In this scenario, a gene replacement therapy for SMA was first introduced in 2010, whereby a self-complementary adeno-associated viral serotype 9 vector carrying a copy of the gene coding for SMN (scAAV9-SMN1) was systemically introduced into the SMNΔ7 mouse model [40–42]. Three pioneering studies demonstrated consistent results that single-dose intravenous (i.v.) injection of scAAV9-SMN1 successfully increased functional SMN protein expression in both CNS and peripheral tissues and resulted in a markedly extended lifespan, as well as improved motor function and NMJ electrophysiology [40–42]. Another study in the same year demonstrated enhanced motor function, improved NMJ architecture, and extended lifespan by delivering an AAV8 vector expressing human SMN directly into the CNS of SMNΔ7 mice [43]. The clinical applications of the scAAV9-SMN1 gene therapy have since been studied extensively, and promising results were reported in 2017. After a single i.v. dose of scAAV9-SMN1 treatment was given to a cohort of type 1 SMA patients, the patients presented better motor function and achieved motor milestones, as well as longer survival, relative to historical cohorts [NCT02122952] [7]. This scAAV9-SMN1 therapy, namely onasemnogene abeparvovec, was approved by the FDA in 2019. Notably, this approval was not affected by one of the pivotal research articles pertaining to the scAAV9-SMN1 gene therapy being retracted in 2022 due to multiple inaccuracies in data reporting [44]. Updates on onasemnogene abeparvovec clinical trials are summarized in Table 2. The drug mechanisms, routes of administration, clinical uses, therapeutic effects, and side effects of three FDA-approved DMTs for SMA are outlined in Table 3, including several meta-analyses demonstrating the effectiveness of these therapies in terms of stabilizing or improving motor function across a diverse population of children, adolescents, and adults with varied SMA phenotypes [45–49]. All three DMTs have demonstrated that presymptomatic treatment initiated following SMA newborn screening leads to significantly better outcomes compared to treatment initiated at the symptomatic stage [50].

**Table 2:** Update on clinical trials for onasemnogene abeparvovec in spinal muscular atrophy (SMA)

Trial name (ID)	Phase/route	Patient recruited	Interventional model	Status	Outcome measure
SMART (NCT04851873)	3b/i.v	Any (symptomatic) SMA type, weighed ≧8.5 kg and ≦21 kg	Multi-center, open label, single group assignment	Completed	Safety, efficacy
STEER (NCT05089656)	3a/i.t	SMA type 2 (able to sit, never walked), aged 2 to ≦18 years	Multi-center randomized, sham-controlled double-blind study, cross over assignment	Ongoing	Safety, efficacy
STRONG (NCT03381729)	1/i.t	SMN2: 3 copies, aged ≧ 6 months and < 5 years	Parallel assignment, open label, non-randomized, multi-center	Terminated	Safety, tolerability
STRENGTH (NCT05386680)	3b/i.t	Discontinued treatment with nusinersen or risdiplam, aged 2 to < 18 years	Open-label, non-randomized, single arm, multi-center study	Ongoing	Safety, tolerability, efficacy
SPECTRUM (NCT05335876)	3/i.v. and i.t	Long-term follow-up of patients with SMA treated with onasemnogene abeparvovec in clinical trials	15-years follow up from the date of onasemnogene abeparvovec administration	Ongoing	Safety, tolerability, efficacy

i.v.: intravenous; i.t.: intrathecal

**Table 3:** The three approved disease-modifying therapies for spinal muscular atrophy (SMA)

Drug (brand name)	Category/mechanism	Usage/route of administration	Dosing frequency	Age restriction	Effects	Side effects
Nusinersen (Spinraza)	Antisense oligonucleotide/alters SMN2 splicing	Fixed dose: 12 mg; intrathecal injection	Six injections in the first years, then three injections per year; lifelong use	No age restriction	Stabilizes or improves motor function in SMA patients of all ages, with better outcomes when treated presymptomatically	Transient deafness, fever, urinary tract infection, coagulation abnormalities, headache, dizziness, back pain, vomiting, and post-lumbar puncture syndrome
Onsemnogene abeparvovec (Zolgensma)	Gene therapy/SMN1 gene carried by AAV9 vector	Dose based on body weight: 1.1 × 10^14^ vg/kg; intravenous injection	One-time treatment	Under 2 years	Stabilizes or improves motor function in SMA patients of < 2 years, with better outcomes when treated presymptomatically	Vomiting, fever, temporarily elevated ALT/AST, hepatoxicity, decreased platelet count, thrombotic microangiopathy, renal impairment, and elevation of troponin I
Risdiplam (Evrysdi)	Small molecule/alters SMN2 splicing	#Dose based on body weight capped at 5 mg once daily for SMA patients aged ≥ 2 years (≥ 20 kg); oral route	Once daily/lifelong use	No age restriction	Stabilizes or improves motor function in SMA patients of all ages, with better outcomes when treated presymptomatically	Fever, rash, mouth ulcers, headache, loose stool, abdominal pain, joint pain, urinary tract infections, and hypoglycemia

ALT: alanine aminotransferase; AST: aspartate aminotransferase

^#^For ages 16 days to < 2 months: 0.15 mg/kg once daily; for ages 2 months to < 2 years: 0.2 mg/kg once daily; for ages ≥ 2 years (< 20 kg): 0.25 mg/kg once daily.

Notably, although SMA was initially considered a motor neuron-centered disease that requires therapeutics targeting the CNS, peripheral organs are also affected by systemic SMN deficiency [51]. Therefore, it is critical to determine the most efficacious route for administering the AAV vector used for treating SMA. In animal studies, both centrally (i.c.v.) and peripherally (i.v.) delivered AAV9-SMN successfully rescued the survival of SMA mice. Induction of SMN expression in the spinal cord was significantly greater when delivered by i.c.v. than by i.v., concomitant with a greater number of motor neurons being preserved in the i.c.v. treated group [52]. In contrast, better NMJ morphology was observed in the i.v. treated group, implying that SMN is needed at the muscle end to help better maintain the NMJ [52, 53]. Interestingly, restricting SMN restoration in the neurons by i.c.v. delivery of AAV9-SMN with a neuron-specific promoter failed to rescue the disease phenotype of SMA mice, reinforcing the idea that non-neuron cell types also play important roles in SMA pathology [54].

Clinically, intrathecal (i.t.) delivery of onasemnogene abeparvovec, evaluated in a phase 1 ascending-dose study involving sitting, nonambulatory patients with SMA, was found to be safe and well-tolerated. In addition, efficacy was demonstrated in SMA patients aged 2–5 years who received the medium dose (1.2 × 10^14^ vg), as evidenced by improvements in HFMSE scores, which exceeded those typically observed for the disease [55]. Altogether, preclinical studies have indicated an advantage of ubiquitous restoration of SMN throughout different organs, supporting that onasemnogene abeparvovec should be delivered systemically via i.v. infusion, though i.t.-based administration of onasemnogene abeparvovec may also benefit patients. However, a detailed study comparing treatment efficacy following central versus peripheral administration is warranted to provide direct evidence of the best administration route.

In addition to gene therapies designed to boost SMN protein production, other preclinical animal studies are being rigorously pursued aimed at uncovering novel indirect or non-SMN disease modifiers as targets for AAV-based gene therapies to treat SMA or to facilitate current SMA treatments. These alternative targets are being sought largely due to some patients proving non-responsive to currently available treatments. Moreover, identifying additional therapeutic targets that could be applied synergistically with current SMN-restoring therapies has the potential to expand the therapeutic window. In the following section, we briefly describe the background of each target, treatment regimens, and outcomes (see also the summary in Table 4).

- AAV-mediated delivery of protein-coding genes other than *SMN1*
- scAAV9-UBA1

**Table 4:** Pioneering preclinical studies on AAV-based gene therapies for treating spinal muscular atrophy (SMA)

Treatment					Mouse model	Survival time (days)	SMN level	Motor function	Physiology				Refs.
Target	AAV type	Route	Dose (vg)	Treatment day(s)					Body weight	Spinal MN	NMJ morphology	Myofiber size	
Protein-coding genes													
* UBA1* (*E1 ubiquitin-like modifier activating enzyme 1*)	scAAV9	i.v.	2.4 × 10^11^	P0	Taiwanese mice	9– > 12	+	+	+	+	+	+	[57]
* TMEM41B* (*Stasimon*; *STAS*)	scAAV9	i.c.v.	1 × 10^11^	P0	SMNΔ7	–	−	+	−	+	−	na	[61]
* PLS3* (*Plastin 3*)	AAV9	i.c.m.	1 × 10^10^	P1	SMNΔ7	11– > 17	−	na	na	na	na	na	[65]
* PLS3*	scAAV9	i.v.	1 × 10^11^	P1	Smn^2B/–^	30– > 43	−	na	-	na	na	na	[66]
					SMNΔ7	–	−	na	na	na	na	na	
* PLS3* w/morpholino ASO					SMNΔ7	30– > 44	+	+	+	na	+	+	
* SNCA* (*Alpha Synuclein*)	scAAV9	i.c.v.	1 × 10^11^	P1	Smn^2B/–^	–	na	na	−	na	na	na	[67]
			3 × 10^11^	P1	Smn^2B/–^	26– > 49	na	na	+	na	+	na	
* STMN1* (*Stathmin*)	scAAV9	i.c.v.	1 × 10^11^	P2	Smn^2B/–^	20– > 30	−	+	+	+	+	na	[72]
* DOK7* (*Downstream of tyrosine kinases 7*)	scAAV9	i.v.	1 × 10^11^	P1	Smn^2B/–^	21– > 22	−	+	−	−	+	+	[82]
Small non-coding RNAs													
miR-23a	scAAV9	i.v.	1 × 10^11^	P1	Smn^2B/–^	20– > 35	−	na	−	+	+	+	[87]
miR-34a	scAAV9	i.v.	1 × 10^10^	P1	SMNΔ7	–	na	+	na	na	+	na	[88]
siPTEN	scAAV6	i.m.	1 × 10^10^	P1	SMNΔ7	na	na	na	na	na	+	na	[91]
	scAAV9	i.v.	1 × 10^10^	P1	SMNΔ7	10– > 30	−	+	+	+	na	na	
ExSpeU1 (Exon-specifically engineered U1 snRNA)	AAV9	i.p.	1.5 × 10^12^	P0 + P2	Taiwanese mice	10– > 219	+	+	+	na	na	na	[93]
Gene editing													
Adenine base editor (ABE; ABE8e-SpyMac)	AAV9	i.c.v.	2.7 × 10^13^ vg/kg body weight	P0	SMNΔ7	17– > 22	+	na	na	na	na	na	[154]
ABE w/nusinersen					SMNΔ7	(17– >) 29– > 77	+	+	+	na	na	na	
SMN1 homology-independent targeted integration (SMN1-HITI)	AAV-PHP.eB	i.v.	1 × 10^11^	P0.5	SMNΔ7	Male: 15– > 18.5; female: 15– > 17	na	+	+	na	na	na	[156]
SMN1-HITI w/ SMN1 cDNA						Male: 72.5– > 182; female: 176– > 220	+	+	+	na	na	na	

MN: motor neuron; i.v.: intravenous; i.c.v.: intracerebroventricular; i.c.m.: intracisterna magna; i.m.: intramuscular; i.p.: intraperitoneal; +: significantly improved; −: no significant change; na: not applicable; Ref.: reference; w/, with

Deficiency of SMN protein in mouse and Drosophila models of SMA has been shown to result in reduced expression of E1 ubiquitin-like modifier activating enzyme 1 (UBA1), which disrupts ubiquitin homeostasis. Interestingly, mutation in the *UBA1* gene is a known cause of X-linked spinal muscular atrophy type 2, a rare SMA subtype that also elicits SMA-like symptoms. Suppression of the conserved *uba1* gene in zebrafish by genetic and pharmacological approaches was observed to result in SMA-like motor neuron symptoms [56]. Moreover, the link between the common type of SMA and disrupted ubiquitin homeostasis is conserved in humans. Powis et al. uncovered that induced pluripotent stem cells (iPSC) from SMA patients that had been differentiated into motor neurons exhibited reduced UBA1 expression levels, indicating that UBA1 may represent a potential therapeutic target for SMA. To further explore the hypothesis that restoring UBA1 levels could rescue the SMA phenotype, Powis et al. administered the “Taiwanese” mouse model of SMA with an i.v. injection of 2.4 × 10^11^ scAAV9-UBA1 viral genomes on the day of birth (postnatal day P0). Compared to mice receiving control scAAV-GFP, the scAAV9-UBA1-treated group presented improved body weight, motor performance, spinal motor neuron numbers, neuromuscular innervation, myofiber size, and heart and liver pathology, and their survival time increased from 9 to 12 days. Additionally, the treatment corrected the disruption to ubiquitin homeostasis at a molecular level [57].

b) scAAV9-STAS

*Stasimon* (*STAS*), also known as *TMEM41B*, is an U12 intron-containing gene encoding an ER-resident transmembrane protein, and U12 splicing was found to be disrupted by SMN deficiency [58, 59]. *STAS* has been linked to SMA, as not only is it regulated by SMN protein, but it is also required for motor circuit development in both Drosophila and zebrafish, with *STAS* overexpression found to reduce SMN deficiency-induced neuronal phenotypes in these organisms [60]. In addition, both misprocessing and reduced expression of *STAS* have been uncovered in pathology-associated neurons in the motor circuits of SMA model mice [60]. To establish how *STAS* dysfunction contributes to SMA pathology, Simon et al. performed scAAV9-mediated *STAS* gene delivery on the SMNΔ7 mouse model by administering an i.c.v. injection of 1 × 10^11^ genome copies of respective viral vectors at P0. The scAAV9-STAS treatment showed rescue effects on motor function, motor neuron survival, and synaptic connections from proprioceptive neurons to motor neurons. However, the treated SMNΔ7 mice exhibited no significant improvements in body weight or lifespan upon scAAV9-STAS treatment [61].

c) scAAV9-PLS3

Plastin 3 (PLS3) is an evolutionarily conserved protein that binds and bundles actin filaments. A study on siblings displaying *SMN1* deletion and with varying disease severities unbiasedly uncovered an association between PLS3 expression levels and SMA severity in female patients. Overexpression of PLS3 not only increased F-actin levels in a HEK293T cell line, but it also rescued axongenesis in vitro of primary motor neurons from Smn^−/−^;SMN2^+/+^ mice, another SMA model, as well as motor neuron outgrowth in vivo of zebrafish subjected to *smn* knockdown [62]. To further elucidate how PLS3 overexpression ameliorates SMA severity in a more relevant disease model in vivo, Ackermann et al. generated the “Taiwanese” SMA mouse model by overexpressing PLS3 fused with a V5 tag (SMA^PLS3V5^) in background strains of varying SMA disease severity. The 50% FVB/N and 50% C57BL/6N mixed backgrounds exhibit less severe disease than the pure C57BL/6N line. In the less severe mixed background SMA mouse lines, PLS3 overexpression rescued body weight, myofiber size, and motor function, in part by improving F-actin dynamics and functional connectivity at neuromuscular synapses, with mean survival time being extended from ~ 17 days to 19 days. However, PLS3 overexpression in SMA mice from the pure C57BL/6N background only improved neuromuscular endplate and myofiber sizes [63]. In addition, other disease-modifying mechanisms independent of F-actin organization also exist. For instance, investigations into functional domains of PLS3 have indicated that PLS3 can interact with Ca^2+^ ions, thereby supporting the growth of Smn-deficient motor neurons in zebrafish [64]. Moreover, selection of PLS3 as a therapeutic target has been further justified by observations that PLS3 expression levels were significantly lower in the spinal cord of SMNΔ7 mice at post-natal day 5 (P5) and P10 than detected in wild type mice at the same stages [65].

Given these findings, two studies have aimed to develop an AAV-based disease-modifying drug for SMA that targets PLS3. Kaifer et al. demonstrated that i.v. delivery of 10^11^ genome copies of scAAV9-PLS3 at P1 extended the survival time of Smn^2B/−^ mice that display intermediate disease severity, but this rescue effect was not seen in severe SMNΔ7 mice subjected to scAAV-PLS3 treatment alone [66]. Nevertheless, the scAAV9-PLS3 treatment significantly ameliorated the disease severity of SMNΔ7 mice when they were co-administered with 2 nmol of a splicing-correcting morpholino ASO. In addition to the rescue effects of the morpholino ASO treatment on SMNΔ7 mice, co-administration with scAAV9-PLS3 improved motor function, neuromuscular endplate morphology, and myofiber size. Moreover, the survival time of these morpholino ASO plus scAAV9-PLS3 co-treated SMNΔ7 mice increased from 30 to 43 days [66]. In contrast to i.v.-administered scAAV9-PLS3 delivery alone neonatally, which did not extend SMNΔ7 mouse survival, delivery of AAV9-PLS3 directly into the CNS via intracisternal magna (i.c.m.) injection proved more efficacious. By administering just 10^10^ vg of AAV9-PLS3 into the CNS of SMNΔ7 mice at P1, median survival time was increased significantly from 11 to 17 days [65]. However, that study did not evaluate how AAV9-PLS3 delivery affected motor ability or neuromuscular physiology.

d) scAAV9-SNCA

Transcriptional profiling of motor neurons displaying differential vulnerability to disease may reveal genetic contributors to motor neuron diseases. One such study compared the differentially-expressed transcripts identified from four independent screening datasets, which uncovered the same transcript patterns between the differentially vulnerable motor neurons across three motor neuron diseases, i.e., SMA, ALS, and spinobulbar muscular atrophy [67]. In total, six gene transcripts were identified as being altered in a common direction from three microarray datasets published previously and their own RNAseq data.

One of the commonly altered genes in that study, *Alpha synuclein* (*SNCA*), was reported to be downregulated in the spinal cord and fibroblasts of SMA patients and it has shown protective effects against degenerative stresses on neuronal cells [68–70]. Therefore, *SNCA* became the first of the six potential therapeutic targets to be investigated further. A dose of either 1 × 10^11^ or 3 × 10^11^ scAAV9-SNCA viral particles was administered via i.c.v. injection into the Smn^2B/−^ mouse model of SMA at P1. Whereas the lower dose of scAAV9-SNCA resulted in no significant impact on survival time or weight gain, the higher dose enhanced median survival time from 26 to 49 days. Moreover, the higher scAAV9-SNCA dose improved the motor function and NMJ morphology of the of Smn^2B/−^ mice compared to the untreated control group [67]. It remains to be explored whether SNCA overexpression protects neurons by reducing stress-related signaling, as observed for stressed conditions, or through other mechanisms [70, 71].

e) scAAV9-STMN1

As the comparative study across four transcriptional screens by Kline et al. showed promising results through its identification of *SNCA* as a functionally relevant disease modifier [67], another investigation was initiated on a further candidate therapeutic target of SMA [72]. Stathmin (STMN1) is a ubiquitously expressed microtubule-binding protein important for regulating microtubule dynamics and it was reported previously as being dysregulated in mouse models of ALS and SMA [73, 74]. Interestingly, *Stmn1* gene deficiency prompts central and peripheral axon degeneration in aging mice [75]. As *STMN1* transcript levels were reduced in the datasets of all four screens [67], Villalon et al. investigated the disease-modifying effects of overexpressing STMN1 in the Smn^2B/−^ mouse model by using scAAV9 as a delivery vector. Administration of 1 × 10^11^ scAAV9-STMN1 viral particles by i.c.v. injection at P2 extended the median survival time of the SMA model mice from ~ 20 to 30 days. In addition, body weight, motor function, motor neuron survival, motor neuron size, NMJ morphology, and microtubule filamentous networks were all improved by the scAAV9-STMN1 treatment [72].

f) scAAV9-DOK7

Downstream of tyrosine kinase 7 (DOK7) is a noncatalytic scaffold protein that mediates activation and signal transduction of the Agrin/MuSK signaling pathway [76]. DOK7 is essential for NMJ development, and *DOK7* mutation has been linked to NMJ synaptopathies [77]. The potential of *DOK7* as a SMA disease modifier is underscored by the emerging significance of its upstream operator, Agrin, in SMA-associated NMJ pathology [78, 79]. Importantly, overexpression of DOK7 has been shown to ameliorate NMJ-linked symptoms of both Emery-Dreyefuss muscular dystrophy and ALS [80, 81]. Using scAAV9-mediated gene delivery, Kaifer et al. examined the potential of DOK7 overexpression to reduce symptoms in Smn^2B/−^ mice [82]. Following a single i.v. dose of 1 × 10^11^ scAAV9-DOK7 viral genomes at P1, median survival was marginally, yet significantly, increased by one day (from 21 to 22 days). Muscle physiology–including myofiber size, grip strength, and NMJ endplate size—was partially restored, but motor neuron numbers and size were not improved by the scAAV9-DOK7 treatment [82].

2) AAV-based delivery of microRNAs (miRNAs) and other small non-coding RNAs

The significance of miRNAs to neurodegenerative diseases such as SMA and ALS is only beginning to emerge [83]. Not only are their expression levels affected during the neurodegenerative process, but they also can actively influence disease progression by regulating cell death, neurite outgrowth, and excitotoxicity [84–86]. Hence, the development of novel miRNA-based gene therapies has garnered attention with regard to facilitating the performance of currently available treatments. Dysregulated miRNAs in SMA contexts represent primary candidates for miRNA-based therapies, as they may potentially contribute to SMA pathology. Screening efforts to identify candidate miRNAs should analyze the molecular mechanisms by which they contribute to SMA pathology to rule out miRNAs that are secondarily altered upon disease onset.

- scAAV9-miR-23a

Kaifer et al. reported that a series of miRNAs were downregulated in iPSC-derived motor neurons of type I/II SMA patients [87]. Among those miRNAs, the authors selected miR-23a for further analyses based on its ability to ameliorate muscular atrophy and neuroprotective (anti-apoptotic and pro-myelinating) effects. Introducing miR-23a into iPSC-derived motor neurons of SMA patients partially rescued the motor neuron loss induced by astrocyte-conditioned media. The disease-modifying efficacy of miR-23a was further examined by delivering it into Smn^2B/−^ mice via the scAAV9 vector. A single i.v. dose of 1 × 10^11^ scAAV9-miR-23a viral genomes at P1 partially alleviated SMA neuromuscular pathology in the Smn^2B/−^ mice, as revealed by increased motor neuron size, NMJ endplate area and innervation, and myofiber cross-sectional area relative to controls. In addition, the scAAV9-miR-23a treatment (both via i.v. and i.c.v. routes) extended median survival from ~ 20 to ~ 35 days, albeit without significant rescue of body weight throughout lifespan [87].

b) scAAV9-miR-34a

Chen et al. demonstrated that, among a list of miRNAs that function in the spinal cord and are enriched during motor neuron and/or interneuron development, miR-34a exhibits the most consistent downregulation during SMA onset and progression both in iPSC-derived motor neurons of type 1 SMA patients and in the spinal cord of SMNΔ7 mice [88]. Moreover, knocking out the miR-34/449 family in non-SMA mice was found to recapitulate SMA pathology at the neuromuscular level, as revealed by swelling of axonal terminals, shrinkage of NMJ endplate areas, and myofiber atrophy. Introducing a single i.v. dose of 1 × 10^10^ scAAV9-miR-34a viral genomes at P0 improved the ability of SMNΔ7 mice to right themselves at P7, which was strongly correlated with restoration of NMJ endplate size. However, no evidence was presented in that study to show if the lifespan of the SMNΔ7 mice was extended by the scAAV9-miR-34a treatment [88].

c) scAAV6/9-siPTEN

Small interfering RNAs (siRNAs) share many characteristics with miRNAs, both being short RNA duplex molecules processed by Dicer and that exert their functions primarily through the formation of an RNA-induced silencing complex. One critical difference distinguishing siRNAs from miRNAs is that the former are highly specific to a single gene target, whereas the latter often have multiple gene targets [89]. Consequently, the development of siRNA-based therapeutics is based solely on the biological roles of its target gene. In contrast, miRNAs may exert their own physiological regulatory roles.

In a previous study, Ning et al. uncovered that PTEN depletion promoted the survival of SMN-deficient motor neurons [90], prompting their subsequent investigation of an siRNA-based SMA therapy targeting PTEN [91]. First, they demonstrated that their siPTEN treatment, involving local injection of a single dose of 10^10^ scAAV6-siPTEN (viral genomes) into the levator auris longus muscle of SMNΔ7 mice at P1, significantly ameliorated innervated NMJ pathology. Next, they systemically delivered siPTEN via i.v. injection of scAAV9 (10^10^ viral genomes) and found that doing so increased the mean lifespan of SMNΔ7 mice from ~ 10 to ~ 30 days relative to the control siRNA-treated group [91]. Importantly, knocking down PTEN also promoted motor neuron survival in vivo [91].

d) AAV9-ExspeU1

Small nuclear RNAs (snRNAs) are another group of small non-coding RNAs that can bind to several proteins to form small nuclear ribonucleoprotein particles (snRNPs), which play a primary role in RNA splicing. One type of snRNP composed of U1 snRNA (i.e., U1 snRNP) is critical for defining exons during the precursor-mRNA splicing process, with disruption of this process potentially causing exon skipping. It has been shown that several disease-causing splicing mutations, including SMN2 exon 7 in the context of SMA, can be corrected by exon-specific engineered U1 snRNAs (ExSpeU1) [92]. As a follow-up study, Donadon et al. systemically delivered a U1 snRNA (ExSpeU1sma) specifically tailored to correct the SMN2 exon 7 splicing defect into the Taiwanese severe SMA mouse model (Smn^−/−^; SMN2^2TG/0^) via two intraperitoneal (i.p.) injections of AAV9-ExspeU1sma (1.5 × 10^12^ vg per mouse) at P0 and P2. This treatment greatly extended the survival of the mice from ~ 10 to ~ 219 days. Rescue effects in terms of body weight, tail length, and motor function were also observed. In addition, by correcting the splicing defect, SMN protein levels at P7 in heart, muscle, liver, and spinal cord, but not in brain, were all significantly increased [93].

In summary, these preclinical studies have generated promising results from various AAV-based gene therapies applied to different SMA mouse models. Although some of these studies uncovered therapeutic effects from increased SMN protein levels, the majority of treatments did not affect SMN protein levels. Interestingly, these SMN-independent approaches also improved neuromuscular physiology, motor function, and even survival time, implying that even symptomatic relief and/or functional compensation of SMN protein could be beneficial in SMA contexts. Hence, it would be valuable to investigate if these SMN-independent AAV-based gene therapies hold promise to be applied in combination with onasemnogene abeparvovec as AAV cocktails.

In addition, it is important to select a suitable serotype of the AAV vector for gene therapy. We have outlined the specific advantages and safety profiles of AAV vectors that have been clinically approved or commonly used in NMD studies in Table 5 [21, 94–102]. Among multiple AAV serotypes, AAV9 has been almost exclusively selected for both animal studies and clinically for SMA treatment via systemic and CNS-targeted routes due to its superior transduction efficiency into cells, its ability to cross the blood–brain-barrier, and its tropism to a wide range of tissues [97]. Selection of AAV serotypes for DMD gene therapy is mainly based on tropism favoring skeletal muscle tissue, thereby making AAV6, AAV8, and AAV9 common choices. Additionally, to reduce the immunogenicity of the AAV capsid, a non-human-originated serotype derived from rhesus monkey, rAAVrh74, is considered clinically superior for DMD treatment, as it also mediates effective transgene expression in muscles [103]. Overall, clinical studies are still required to confirm the safety and efficacy of each therapeutic gene target and the potential of new AAV serotypes for real patients.

**Table 5:** Tropism, advantages and safety concerns of clinically approved or NMD-relevant AAV serotypes

Serotype	Tropism towards the CNS and/or muscle	Key advantages	Safety concerns	FDA-approved drugs (brand name; year)/disease
AAV2	Both	Well-studied, safe for retina and CNS	Prevalence of neutralizing antibodies, immune activation	Voretigene neparvovec-rzyl (Luxturna; 2017)/biallelic RPE65 mutation-associated retinal dystrophy
AAV5	Both	Lower immune reactivity	Dose-related liver toxicity	Etranacogene dezaparvovec-drlb (Hemgenix; 2022)/hemophilia B Valoctocogene roxaparvovec-rvox (Roctavian; 2023)/adults with severe hemophilia A
AAV6	Muscle	Effective for muscle transduction	Mild immune activation	na
AAV8	Both	Robust liver and muscle transduction	Liver toxicity at high doses	na
AAV9	Both	Crosses BBB, CNS and muscle targeting	Cardiac toxicity, immune activation	Onasemnogene abeparvovec (Zolgensma; 2019)/spinal muscular atrophy aged less than 2 years
rAAVrh74	Muscle	Reduced immunogenicity, effective for muscle transduction	Risk of myocarditis	Delandistrogene moxeparvovec-rokl (Elevidys; 2023)/ambulatory and non-ambulatory individuals 4 years of age and older with DMD with a confirmed mutation in the DMD gene

BBB: blood–brain barrier; CNS: central nervous system; na: not applicable

## Gene therapy for DMD: complemented by RNA and small molecule therapies for patients of limited age

Duchenne muscular dystrophy (DMD) is a life-threatening X-linked recessive disease and the most common genetic neuromuscular disorder. It is caused by mutations in the *DMD* gene, which result in the absence or insufficient levels of functional dystrophin. Without dystrophin, muscles are more vulnerable to damage, leading to progressive muscle weakness and dysfunction. This deterioration ultimately causes loss of ambulation, cardiomyopathy, respiratory insufficiency, and can be fatal [104, 105]. The first symptoms of DMD include difficulty climbing stairs, a waddling gait, and frequent falls, typically appearing around 2 to 3 years of age. By 10 to 12 years old, most patients become wheelchair-dependent, and assisted ventilation is usually required by age 20. Despite optimal care, most individuals with DMD succumb to cardiac and/or respiratory failure between 20 and 40 years of age [105]. Mutations in the *DMD* gene can also lead to Becker muscular dystrophy, a milder form of the disease characterized by later onset and slower progression compared to DMD [106]. The estimated global incidence of DMD is 1 in 3,500 to 5,000 live male births [107].

Advanced multidisciplinary care and steroid treatments have improved DMD patient survival. On this basis, the FDA has recently approved two drugs that target the anti-inflammatory pathogenic processes of DMD. Vamorolone (October 2023), a novel corticosteroid that acts through the glucocorticoid receptor to exert anti-inflammatory and immunosuppressive effects, is believed to be better-tolerated in terms of side effects than current standard-of-care corticosteroids [108]. Givinostat (March 2024), a histone deacetylase inhibitor, is the first nonsteroidal drug for all DMD genetic variants, which acts by reducing inflammation and preventing muscle loss [109]. As prolonged survival becomes more common, various anticipatory diagnostic and therapeutic strategies are increasingly being adopted [110, 111].

Recent studies have significantly enhanced our understanding of the primary and secondary mechanisms underlying DMD. This improved insight into pathogenesis is driving the development of innovative DMTs of DMD [112]. To date, several treatments designed to restore the missing dystrophin protein through gene-based therapeutic strategies have been approved by the FDA. Using predesigned ASOs, mutant codons can be targeted to induce exon skipping of the DMD gene, allowing for production of proteins having partial dystrophin functionality. Approved therapies include eteplirsen for exon 51 skipping [15], golodirsen and viltolarsen for exon 53 skipping [16, 17], and casimersen for exon 45 skipping [18] (Table 1). In terms of AAV-based gene replacement therapy for DMD, the large full-length *DMD* gene presents difficulties for AAV vectors for gene delivery. As a solution, a smaller, functional micro-dystrophin gene is being used instead. An update on recent progress in the development of micro-dystrophin gene replacement therapies to treat DMD is summarized in Table 6 [113, 114]. In June 2023, the FDA granted approval for delandistrogene moxeparvovec, a gene therapy targeting DMD, specifically for ambulatory patients aged 4 to 5. In June 2024, this approval was expanded to include both ambulatory and non-ambulatory patients aged 4 years and older [8, 115]. These strategies, which focus on prevention, early identification, and treatment of predictable and potentially modifiable disease complications, aim to provide a better quality of life for DMD patients and their families.

**Table 6:** Update on developments of micro-dystrophin gene replacement therapy for Duchenne muscular dystrophy (DMD)

Drug name/Trial ID	Sponsor	Micro-dystrophin	AAV serotype	Promoter	Status
Delandistrogene moxeparvovec (SRP-9001)/ NCT05096221 NCT03375164	Sarepta Therapeutics	ΔR4-23/ΔCT	rAAVrh74	MHCK7	FDA Approval/ Phase III Phase I/II
PF-06939926/ NCT04281485 NCT03362502	Pfizer	ΔR3-19/20–21/ΔCT	AAV9	hMSP	Terminated Phase III* Phase Ib#
SGT-001/ NCT03368742 SGT-003/ NCT06138639	Solid Biosciences	ΔR2-15/R18-22/ΔCT	AAV9 AAVSLB101	CK8	Phase I/II Phase I/II
RGX-202/ NCT05693142	Regenxbio	ΔR4-23 (includes CT)	AAV8	Spc5-12	Phase I/II

CT: C-terminal domain

^#^The death of a 16-year-old non-ambulatory trial participant with advanced disease, who was treated with a high dose (2 × 10^14^ vg/kg) of PF-06939926 in an open-label Phase Ib trial (NCT03362502), led to a temporary FDA hold on the drug [113]

^*^Pfizer Inc. have announced that its Phase 3 trial (NCT03362502) of the mini-dystrophin gene therapy in young boys with DMD did not achieve its primary goal of improving motor function [114]

Although the FDA has approved four ASOs and one AAV-mediated gene replacement therapy for DMD, these current therapies are not sufficiently curative and only primarily aim to slow down disease progression, and their overall impact remains limited. Specifically, the genetic mutations causing DMD are highly variable and unique to individual patients, which restricts the number of patients who benefit from existing ASO therapies [116]. To address this limitation, multi-exon skipping has emerged as a promising strategy. Unlike the single-exon approach of current ASOs, this method employs an ASO "cocktail" to simultaneously skip multiple exons. For instance, a cocktail targeting exons 45–55 has been validated in both in vitro and in vivo studies, offering the potential to treat nearly 50% of DMD patients [117].

AAV-based gene replacement therapies have also proved challenging due to the packaging capacity of AAV vectors, which is limited to approximately 4.7 kilobases. This capacity is insufficient for delivering large coding sequences such as DMD’s 14-kilobase messenger RNA. In addition, the net clinical benefit of delandistrogene moxeparvovec has been questioned [118]. Intriguingly, a novel gene replacement technique leveraging protein trans-splicing mediated by split inteins has demonstrated success in expressing large dystrophins in striated muscles of a DMD mouse model [119]. Restoration of large or full-length dystrophin proteins can be achieved by systemic delivery of AAV vectors, resulting in significant physiological improvements (e.g., less muscle wastage, reduced muscle fibrosis, and increased force production) in dystrophic mice. Importantly, the rescue effects were mediated by a novel type of AAV vector, AAVMYO, administered at low doses, and the improvements were significantly greater when compared to a micro-dystrophin-treated group [119].

In summary, these findings justify continued investigation into the development of novel, enhanced ASO and AAV-based gene replacement therapies for future clinical applications. Another category of AAV-based gene therapy, CRISPR-based gene editing, also known as "myoediting" in DMD contexts, holds promise for providing a one-time treatment for DMD by directly correcting genetic mutations and restoring normal gene expression [120]. As discussed in the next section, although none of these gene editing therapies have been approved for clinical use to treat DMD, several gene editing strategies in preclinical models have demonstrated therapeutic efficacy and safety, paving the way for a potential breakthrough in DMD treatment.

## Unveiling gene therapy breakthroughs: exploring gene editing

Breakthroughs in gene-editing therapy based on CRISPR/Cas9 technology, by which targeted changes can be introduced into the host genome, have raised hope for permanent cures for genetic NMDs by correcting the underlying genetic mutations or modifying gene expression. As exemplified by efforts to develop DMD treatments, robust preclinical studies have been conducted over the past decade on gene-editing approaches, with various editing strategies being deployed to tackle different mutations in the *DMD* gene to restore functional expression. However, several challenges must be addressed, including optimizing gene editing, effectively delivering the gene-editing components to all muscles in the body, and suppressing potential immune responses to the CRISPR therapy [120]. Below, we summarize the main concept and advantages of different CRISPR-mediated gene editing strategies to treat DMD and, in Table 7, we outline several promising preclinical studies on DMD mouse models utilizing systemic delivery via the AAV vector.

**Table 7:** Preclinical studies on systemic-administered, AAV-based gene editing therapies for treating Duchenne muscular dystrophy (DMD)

Gene editing strategy and targeted exons and effects on *DMD* gene	Mouse model	AAV Treatment				Rescued muscles	References
		Serotype	Route	Total dosage per mouse	Nuclease:guide RNA ratio		
Double-cut editing							
Exon 23 excision	mdx	AAV9	i.p.; i.v	2 × 10^14^ vg/ml—30 µl for i.p.; 90 µl for i.v	2:1:1	TA, heart	[123]
Exon 23 excision	mdx	AAV8	i.p.; i.v	5.6 × 10^11^ vg for i.p.; 5.4 × 10^12^ vg for i.v	1:1	Abdominal, diaphragm, heart, TA	[124] [125]
Exon 23 excision	mdx	AAV9	i.p.; i.v	3 × 10^12^ vg for i.p.; 3.6 × 10^12^ vg for i.v	1:1	Heart, gastroc, TA, diaphragm, abdominal, triceps, quads,	[128]
Exon 23 excision	mdx	AAV9	i.v	1.08 × 10^13^ or 4 × 10^14^ vg	2:1 or 1:3	Heart, quads, gastroc	[122]
Exon 21–23 excision	mdx/Utr^+/−^	AAVrh.74	i.v	1 × 10^12^ vg	Single vector	Heart	[129]
Exon 21–23 excision	mdx	AAVrh.74	i.p	1 × 10^12^ vg	Single vector	Heart	[126]
Duplicated exon 18–30 excision (targeting exon 21)	Dup18-30	AAV9	i.v	3 × 10^12^ vg	Single vector	Heart, TA, triceps, diaphragm	[127]
Formation of hybrid exons 47 & 58	Δ52h*DMD*/*mdx*	AAV9	i.v	7.5 × 10^13^ vg/kg	1:1	Heart	[121]
Exon 52–53 excision	mdx^4cv^	AAV6	i.v	1.4 × 10^13^ vg	5:2	TA, diaphragm, soleus, gastroc	[130]
Single-cut editing							
Exon 45 skipping/reframing	ΔEx44	AAV9	i.p	3 × 10^14^ or 5.5 × 10^14^ vg/kg	1:5 or 1:10	Heart, TA, diaphragm	[133]
Exon 45 skipping/reframing	ΔEx44	scAAV9	i.p	8.4 × 10^13^–1.6 × 10^14^ vg/kg	Ranging from 1:0.05 to 1:1	Heart, TA, triceps, diaphragm	[134]
Exon 51 skipping/reframing	ΔEx50	AAV9	i.p	6.3 × 10^10^ vg	n/a	Heart, triceps, gastroc, plantaris, quads, diaphragm	[132]
Exon 51 skipping/reframing	ΔEx50-Dmd-Luc	AAV9	i.p	3 × 10^14^ vg/kg	1:2	Heart, TA, triceps, diaphragm	[137]
Exon 51 skipping/reframing	ΔEx50	AAV9	i.p	2 × 10^14^ or 4 × 10^14^ vg/kg	Single vector	Heart, TA, triceps, diaphragm	[136]
Exon 51 skipping/reframing	ΔEx50;h51KI	AAV9	i.p	1.6 × 10^14^ vg/kg	1:1	Heart, TA, triceps, diaphragm	[135]
Exon knockin							
Insertion of exon 52 or superexon 52–79	Δ52h*DMD*/*mdx*	AAV9	i.v	8.64 × 10^11^ or 7 × 10^11^ vg	1:1 or 1:5	Heart	[142]
Base editing							
CBE, exon 4 skipping	Dmd^E4*^	AAV9	i.p	1.1 × 10^12^ vg	1:1	Heart, TA, diaphragm, quads	[148]
ABE, exon 53 mutation correction	mdx^4cv^	AAV9	i.v	1 × 10^14^ vg/kg	1:2 (in split constructs)	Heart, gastroc, diaphragm	[151]
ABE, exon 45 skipping	ΔEx44	AAV9	i.v	1.5 × 10^14^ & 3 × 10^14^ vg/kg	1:1	Heart, TA	[149]
ABE, exon 50 skipping	ΔEx5051;h50KI	AAV9	i.p.; i.v	1 × 10^14^ vg/kg	1:3 (in split constructs)	Heart, TA, diaphragm	[150]

TA: tibialis anterior muscle; quads: quadriceps muscle; gastroc: gastrocnemius muscle; i.v.: intravenous route; i.p.: intraperitoneal; CBE: cytosine base editor; ABE: adenine base editor

Double-cut exon excision to edit the *DMD* gene involves using two single-guide RNAs (sgRNAs) to target and remove specific exons from the dystrophin gene. This approach can be applied to most DMD cases and is particularly effective for correcting exon duplications. By excising the mutant exons, this method restores the reading frame and allows for the production of functional dystrophin protein, either full-length or truncated. Several studies in which dystrophin function was successfully restored via double-cut gene editing by means of systemic delivery of AAV in DMD mouse models are summarized in Table 7 [121–130]. However, the double-cut method has limitations, including low editing efficiency and off-target effects, as it requires precise and simultaneous cuts at two genomic sites, followed by accurate rejoining of the DNA. This process can be especially challenging across large genomic regions [125]. In addition, using two sgRNAs at the same time increases the risk of unintended genome modifications at the double-strand break (DSB) sites, including DNA inversions, aberrant splicing, or integration of the exogenous DNA from AAV [125]. These unpredictable outcomes pose substantial barriers to the clinical application of this technique [120, 131].

Single-cut gene editing represents a promising strategy for correcting diverse mutations in the *DMD* gene. Using a single sgRNA to target regions near intron–exon boundaries or splice signal sequences, it introduces a single DSB, which is then rejoined via the non-homologous end joining (NHEJ) mechanism. This repair process can result in two desired outcomes, exon skipping or exon reframing, by disrupting splice sites of out-of-frame exons or by restoring the reading frame, respectively. Both outcomes can lead to the restoration of functional dystrophin proteins. Importantly, this method has advantages, such as higher editing efficiency, reduced off-target effects, and minimal genomic alterations compared to double-cut strategies, making it more suitable for correcting DMD mutations [120]. In Table 7, we summarize several studies that successfully restored dystrophin function via single-cut gene editing through systemic delivery of AAV into DMD mouse models [132–137]. Limitations of single-cut gene editing include variable efficiency, which is dependent on the mutation and target site, with the dystrophin restoration rate ranging from 36 to 60% depending on the target exon [131, 138]. In addition, though using only one sgRNA reduces off-target effects, generation of DSBs still poses the potential risk of unintended DNA integration. These challenges highlight the need for careful design and delivery strategies to maximize therapeutic efficacy while minimizing potential adverse effects.

Targeted gene insertion, or exon knockin, is theoretically useful for addressing mutations in critical regions of the *DMD* gene to restore full-length dystrophin protein. The homology-directed repair (HDR) method may achieve this purpose by using a natural DNA repair mechanism, which relies on a donor DNA template to correct the gene precisely. However, HDR is highly dependent on active cell division, making it inefficient in non-dividing, post-mitotic cells such as mature myofibers [131, 139, 140]. In addition, the size of the donor DNA template is limited and dependent on the delivery vectors, with the risk of inverted integration further restricting the clinical applicability of this method, especially for large deletions [140]. In contrast, NHEJ-based homology-independent targeted integration (HITI) represents an alternative approach that circumvents the limitations of HDR. By using CRISPR/Cas9 technology to introduce cuts in both the genome and donor DNA template, the NHEJ repair pathway allows more precise integration of the donor sequence at the target site [131, 140, 141]. Unlike HDR, NHEJ-based HITI works efficiently in both dividing and non-dividing cells, ensuring its applicability in muscle. HITI has been applied successfully in a DMD mouse model to restore dystrophin expression in skeletal and cardiac muscles by inserting the missing exon 52 or a 52–79 superexon [142], as summarized herein in Table 7. Although the rate of restoration in cardiac muscle is variable and with scope for improvement, this method demonstrates potential for treating large-scale deletions and for being applied to over 20% of global DMD patients [142].

The base editing system represents a precise and efficient approach to correcting genetic mutations without relying on the error-prone repairment of DNA DSBs. Two primary types of base editors have been used for this purpose, i.e., cytosine base editors (CBEs) and adenine base editors (ABEs) that mediate C:G-to-T:A and A:T-to-G:C base pair conversions, respectively. The base editors are particularly useful for addressing point mutations in the dystrophin gene by directly repairing the mutation or inducing exon skipping by modifying splice sites [143, 144]. CBEs have been associated with off-target effects at both genomic and transcriptomic levels, raising concerns about unintended consequences, such as oncogenesis, whereas ABEs have been highlighted for their relatively higher specificity and lower off-target activity [131, 145–147]. In Table 7, we present a summary of several studies in which dystrophin function has been rescued via base editing using systemic AAV delivery in DMD mouse models [148–151]. The relatively large size of base editors poses a challenge for delivery using AAV vectors. This issue can be overcome by using a split intein-based approach, which allows efficient assembly of full-length base editors [150, 151]. Additionally, the therapeutic range of base editing is currently limited to specific mutation types, making it suitable for an estimated 25–35% of DMD patients with point mutations [140].

Although CRISPR-based, AAV-mediated gene editing therapies for DMD have been studied extensively over recent years, only one of the various in vivo gene-editing strategies is currently undergoing a clinical trial. A human DMD exon 50 skipping cytosine base-editing drug just entered the recruiting phase in July 2024 (NCT06392724) [152].

Unlike for DMD, no gene-editing therapy for SMA has yet entered clinical trials. Limitations of currently approved SMA drugs, which either require repeat administration or display reduced efficacy over time, have prompted accelerated research into gene-editing therapies. Given that two approved *SMN2* splicing modifiers can restore SMN protein levels and effectively treat SMA, similar concepts have been applied to gene-editing therapies. Specifically, CRISPR/Cas9 technology has been used to correct *SMN2* splicing [153]. However, the first preclinical studies on AAV-based gene-editing therapy to treat SMA in animal models were not published until 2023 [154].

To develop a more desirable permanent therapy that restores endogenous SMN expression with a single dose, while preserving native transcriptional and translational regulatory processes, Arbab et al. performed conversion in vivo of the *SMN2* gene into *SMN1* by base editing using an AAV9 vector [154]. First, candidate base-editing strategies were tested in embryonic stem cells from the SMNΔ7 mouse model. After testing a total of 79 base-editing and nuclease strategies targeting different regions of *SMN2*, one ABE strategy displaying high target efficiency and specificity was selected for further investigation in vivo. A dose of 2.7 × 10^13^ vg/kg body weight of AAV9-ABE was administered via i.c.v. injection into SMNΔ7 mice at P0, which resulted in a conversion rate of 87% C6T (C-to-T exchange at position 6 in exon 7 of SMN2) in GFP-sorted transduced cells labeled by co-delivered AAV9-GFP. This AAV9-ABE treatment alone rescued the motor unit numbers and muscle action potentials of SMNΔ7 mice, with median survival time increasing from 17 to 22 days. Since SMN protein accumulation after AAV9-ABE treatment is relatively slow and thus exceeds the short therapeutic window for SMNΔ7 mice, the authors co-administered nusinersen to attenuate disease progression and extend the therapeutic window. Interestingly, this combination therapy of AAV9-ABE plus nusinersen outperformed nusinersen treatment alone in terms of extending median survival time (from 29 to 77 days) and rescuing motor function [154]. Another study by Alves et al. employing a similar base-editing approach achieved comparable results in vitro in restoring SMN levels by reversing SMN2 exon 7 mutation, further strengthening the feasibility of the ABE-based strategy [155].

**Fig. 1:**
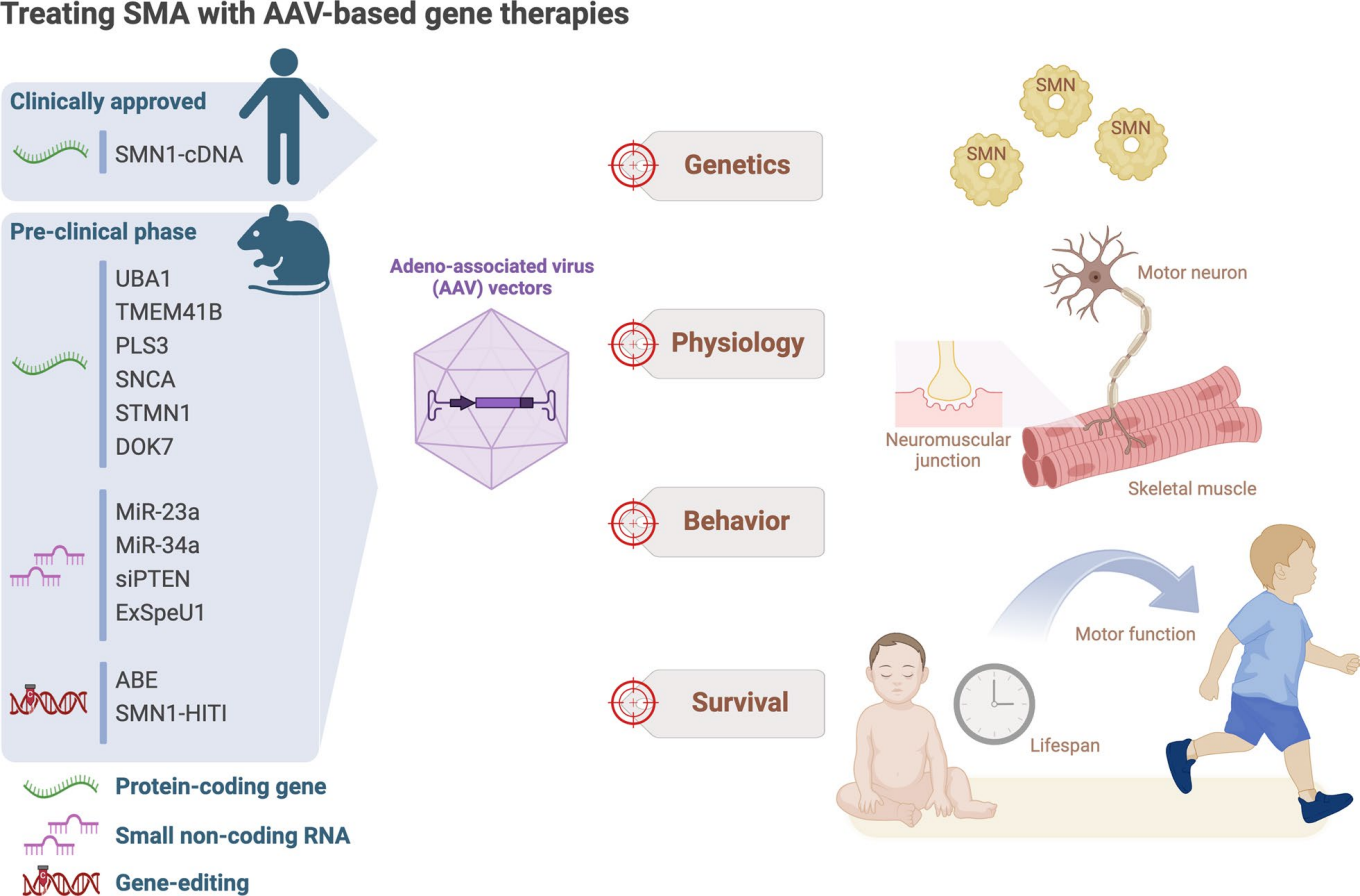
**Illustration of AAV-based gene therapies for treating SMA.** Different genetic materials delivered through AAVs are aimed at benefitting SMA patients with regard to different aspects of disease pathology.

In addition to ABE therapy, a recent study demonstrated promising therapeutic results following knock-in of the *Smn1* gene into the genome of a SMA mouse model through NHEJ-based HITI mechanism [156]. Although HITI-mediated *Smn1* gene knock-in alone only increased the survival time of SMNΔ7 mice by 2–3 days, it exhibited similar effects to onasemnogene abeparvovec therapy in terms of restoring motor function. Furthermore, when HITI-mediated *Smn1* gene knock-in was combined with onasemnogene abeparvovec, survival time increased significantly relative to onasemnogene abeparvovec therapy alone [156]. As highlighted in that study, not only could the NHEJ mechanism be effective even in non-dividing cells such as motor neurons, but the AAV-PHP.eB vector deployed may enhance treatment efficacy by enabling more robust transduction into spinal motor neurons [156]. Together, these gene-editing strategies have shown promise in facilitating currently available SMA drug treatments and even as stand-alone therapy. However, clinical trials are still urgently needed to confirm their applicability and safety in humans. A summary of all of the aforementioned AAV-based gene therapies for treating SMA, including clinically-approved and solely preclinically assessed ones is presented in Fig. 1.

## Pioneering future applications: biomarkers

The concept of precision medicine is now widely recognized. Clinically, a biomarker is often helpful to attain efficacious personalized treatment regimens, not only to optimize treatment dose, frequency, and even combination therapies, but also to predict treatment responses. For instance, dosing frequency for AAV-based gene therapies is limited, primarily due to the immunogenicity of the AAV capsid. Treated individuals may have pre-existing immunity because of prior natural exposure to AAVs, leading to the presence of neutralizing antibodies. More importantly, after the first dose of AAV-based therapy, the immune system mounts a strong humoral and cellular response against the viral capsid, generating neutralizing antibodies and memory T cells that can rapidly neutralize the vector upon re-administration [94, 157, 158]. Therefore, repeat dosing of the genetic materials using the AAV vector would not be feasible without accessory immunomodulating agents. Consequently, precise prescription of the first dose of an AAV-based treatment plan is especially critical. Thus, implementing predictive biomarkers within treatment plans is a desirable strategy for facilitating genetic NMD therapies.

Taking SMA as an example, since patient responsiveness to current therapeutics can vary among individuals, it is difficult to predict efficacy and optimize the choice of treatment plans. The copy number of *SMN2*, the sole gene source of SMN protein for SMA patients, is commonly used to predict age of disease onset, survival rate, and phenotypic severity. However, *SMN2* copy number it is not a perfect indicator because: 1) not all SMN2 copies are equivalent; 2) SMN-independent genetic modifiers exist; and 3) peripheral tissues are also important contributors to SMA pathology [39, 53, 159, 160]. Consequently, it remains important to identify reliable biomarkers that facilitate accurate diagnoses, prognoses, and treatment prescriptions at the earliest time point with the lowest cost.

A good biomarker should be easily detectable and reflect real pathology rather than just secondary symptoms. Studies aimed at identifying biomarkers reflecting SMA disease severity and treatment responses are being rigorously pursued, but evidence supporting the clinical applicability of canonical candidate biomarkers remains equivocal [161]. For example, SMN-related biomarkers appear to be too variable among patients to present any power in predicting disease severity. Moreover, although phosphorylated neurofilament heavy chain (pNfH) represents a promising reliable biomarker of disease severity and treatment efficacy in infant SMA patients, the results are contradictory for adult patients [161]. On the other hand, microRNAs (miRNAs), a class of small non-coding RNAs that regulate gene expression by inducing mRNA degradation or altering translation efficiency, represent promising candidate biomarkers of SMA. This is owing to miRNAs: 1) playing significant roles in motor neuron development and disease; 2) being stably present and easily detectable in biofluids (e.g., blood and cerebrospinal fluid); and 3) often exhibiting tissue- or cell type-specific expression, as is dysregulation of miRNA expression in various disease contexts [83, 86, 88, 162].

Uncovering disease predictive biomarkers can be especially challenging, but it is especially important for neurodegenerative disorders that affect the CNS, as neuronal alterations are more likely to be detectable centrally (e.g., in the cerebrospinal fluid) rather than peripherally (e.g., in blood) [163]. To date, a suite of miRNAs known to be dysregulated in SMA has demonstrated potential as either prognostic or predictive SMA biomarkers, as summarized in Table 8 [88, 163–170]. Fortunato et al. presented a review of miRNAs demonstrating potential as DMD biomarkers for diagnosis, prognosis, and disease progression monitoring [171]. Notably, miRNAs play an active role in cell–cell communication, with some being packaged for exocytosis and secreted as extracellular vesicles (EVs) [88, 172–174]. The EVs containing miRNAs can be taken up by recipient cells, where they may modulate target gene expression [174]. In this scenario, it is possible that a certain panel of miRNAs carried by EVs could represent candidate biomarkers to reflect disease progression and treatment outcome prediction. However, further investigation is needed to determine if EV-derived miRNAs provide greater specificity and sensitivity in prognosis and prediction than the total miRNA from bodily fluids. To date, none of the reported miRNAs among these studies has been tested in terms of predicting the treatment efficacy of any AAV-based gene therapies. Hence, clinical data regarding combined use of AAV-based gene therapies and predictive biomarkers are urgently needed.

**Table 8:** MiRNA biomarkers studied using spinal muscular atrophy (SMA) patient samples

MiRNA	Prognostic or predictive	Association with SMA pathology	Number of subjects (n)	Body fluids	Measurements and changes	Refs.
miR-9, miR-132, miR-206	Prognostic	Differential expression of all three miRNAs in spinal cord, skeletal muscle and serum samples in SMA mice, with presymptomatic changes in serum	Type 2 & 3, n = 6 & 4	Serum	qPCR; upregulation, no correlation with functional outomes was found	[165]
miR-133a	Prognostic/predictive (patients w/nusinersen treatment)	Muscle-enriched miR (myomiR) decrease w/ disease progression	Type 2/3, n = 21	Serum	qPCR; down-regulation, predicts clinical improvement	[163]
miR-206	Prognostic	MyomiR increase in SMA muscle	Type 2 & 3, n = 17 & 6	Serum	qPCR; upregulation	[168]
miR-181a-5p, miR-324-5p, miR-451a	Prognostic	Differentially expressed in SMA muscle	Type 1 & 2, n = 1 & 9; then Type 1, 2, 3, & 4, n = 3, 21, 26, & 1	Serum	miRNAseq, qPCR; up/down-regulation; correlation with phenotypic severity	[164]
miR-146a	Prognostic (patients w/nusinersen treatment)	Upregulated in SMA iPSC-derived astrocytes; increased expression in nusinersen-treated CSF samples	Type 1, 2, & 3, n = 4, 5, & 3	CSF	qPCR array, qPCR; upregulation in functional responders	[169]
miR-107, miR-142-5p, miR-335-5p, miR-660-5p, miR-378a-3p, miR-23a-3p,	Prognostic/predictive (patients w/nusinersen treatment)	Dysregulated levels in plasma between SMA patients and controls	Type 2 & 3, n = 10 & 10	Plasma	miRNAseq, qPCR; up/down-regulation; correlation with functional outcomes	[170]
miR-206 and miR-133a-3p	Prognostic/predictive (patients w/nusinersen treatment)	Differentially expressed between responders and nonresponders	Type 2/3, n = 45	CSF	miRNAseq, qPCR; up/down-regulation; correlation with functional outcomes	[167]
miR-7-5p, miR-15a-5p, miR-15b-3p/5p, miR-126-5p, miR-128-2-5p and miR-130a-3p	Prognostic (patients w/ nusinersen treatment)	Predicted target genes involved in neurogenesis, neuronal differentiation, and growth	SMN2 copy = 2/3, n = 6	CSF	miRNAseq; downregulation	[166]
miR-34a	Prognostic/predictive (patients w/nusinersen treatment)	Enriched in motor neurons at embryonic and neonatal stages; downregulated in spinal cord of SMA mice and human SMA iPSC-derived motor neurons; miR-KO mouse model manifests SMA phenotype; decreased in nusinersen-treated patient CSF samples	Type 1, n = 7	CSF	qPCR; down-regulation, predicts clinical improvement	[88]

w/: with; KO: knockout; CSF: cerebrospinal fluid; iPSC: induced pluripotent stem cells

## Conclusions

In this mini-review, we have covered current successes in gene therapies for treating genetic NMDs. Specifically, in addition to splicing-modifying drugs for SMA, several clinical trials are still ongoing for the only currently approved AAV-based gene replacement therapy, i.e., onasemnogene abeparvovec. Moreover, several preclinical studies have already underscored the potential of a wide range of AAV-based gene therapies to mitigate the severity of SMA. Whether these novel therapies may be applied to strengthen current treatment regimens necessitates more data from clinical settings. Herein, we have also explored the current status of gene therapies for treating DMD, briefly updating on recent progress and/or outcomes of clinical trials of *DMD* gene replacement therapy.

To exemplify breakthroughs in gene therapy attributable to CRISPR/Cas9 technology, we have specifically highlighted two AAV-based gene-editing therapies applied to treating an SMA mouse model. The first one utilized an adenine base-editing strategy to convert the *SMN2* gene into the *SMN1* gene, which alone only elicited a moderate rescue effect. However, synergistic administration together with a one-time nusinersen treatment mitigated SMA symptoms and significantly extended lifespan. The second study took advantage of the HITI approach, which similarly elicited a significant extension in the lifespan of SMA mice when co-administered with the *Smn1* gene replacement therapy. In terms of DMD, a number of gene-editing strategies targeting different disease-causing mutations have been reported. These breakthroughs illuminate the possibility of patients receiving a permanent cure from just a single dose. In addition, they also raise the potential of tackling multiple aspects of NMDs through an AAV cocktail. Despite these advances in AAV-based gene therapy, pioneering studies are still needed to identify valid and informative biomarkers that can guide treatment decisions and predict outcomes. Overall, the groundbreaking development of AAV-based therapeutic approaches has paved the way for future research aimed at curing genetic NMDs.

## Data Availability

Not applicable.

## Abbreviations

AAV: Adeno-associated virus
ABE: Adenine base editor
ALS: Amyotrophic lateral sclerosis
ASO: Antisense oligonucleotide
BCL11A: B-cell lymphoma/leukemia 11A
CNS: Central nervous system
CSF: Cerebrospinal fluid
CT: C-terminal domain
DMD: Duchenne muscular dystrophy (or Dystrophin)
DMT: Disease-modifying therapy
DOK7: Downstream of tyrosine kinase 7
ExSpeU1: Exon-specific engineered U1 small nuclear RNA
FAP: Familial amyloid polyneuropathy
GFP: Green fluorescent protein
HITI: Homology-independent targeted integration
iPSC: Induced pluripotent stem cell
i.c.m.: Intracisterna magna
i.c.v.: Intracerebroventricular
i.m.: Intramuscular
i.t.: Intrathecal
i.ves.: Intravesical
NMD: Neuromuscular disease
NHEJ: Non-homologous end joining
NMJ: Neuromuscular junction
PLS3: Plastin 3
RNAi: RNA interference
SCD: Sickle cell disease
SMA: Spinal muscular atrophy
SMN: Survival motor neuron
SNCA: Alpha synuclein
STAS: Stasimon
STMN1: Stathmin
TDT: Transfusion-dependent β-thalassemia
TMEM41B: Transmembrane protein 41B
TTR: Transthyretin
UBA1: E1 ubiquitin-like modifier activating enzyme 1
vg: Viral genome
